# Campylobacter-Associated Myocarditis in a 17-Year-Old Male

**DOI:** 10.7759/cureus.68326

**Published:** 2024-08-31

**Authors:** Stephen C Ream, Jaclyn Giafaglione, Ana Quintero, Monica Ardura, Stephen Hart

**Affiliations:** 1 Internal Medicine and Pediatrics, The Ohio State University Wexner Medical Center, Columbus, USA; 2 Internal Medicine and Pediatrics, Nationwide Children's Hospital, Columbus, USA; 3 Cardiology, Nationwide Children's Hospital, Columbus, USA; 4 Infectious Disease, Nationwide Children's Hospital, Columbus, USA

**Keywords:** nsaid, chest pain, cardiac mri, myocarditis, campylobacter infection, med-peds, campylobacter enteritis myocarditis

## Abstract

Chest pain is a common presenting complaint in adolescent patients. Myocarditis is an important and potentially serious etiology of chest pain for clinicians who care for these patients to recognize. Myocarditis is commonly virally mediated, while extra-intestinal cardiac manifestations of bacterial enteritis, such as *Campylobacter *infections,are rare. Awareness of this uncommon, but potentially life-threatening pathophysiology is important for clinicians to understand.

In our case, a 17-year-old male presented with chest discomfort, chest pain on inspiration, headache, myalgias, vomiting, and diarrhea. He denied recent viral illnesses or immunizations. He lived in rural Ohio, swam recently in a freshwater lake, and had eaten home-prepared deer meat. His father had diarrhea as well. Presenting vital signs were within normal limits for age. The patient was obese (BMI 48.5), with an otherwise normal physical exam, including a thorough cardiopulmonary assessment. Laboratory workup revealed leukocytosis (16.1 x 10^9^/L) and elevated high-sensitivity troponin (15,857 ng/L, >22,000 ng/L three hours later, ref range <20). Gastrointestinal polymerase chain reaction (PCR) panel detected *Campylobacter *spp., and stool culture was positive for *Campylobacter jejuni*. ECG, echocardiography, chest X-ray, and CT angiography were normal. Cardiac MRI revealed an increased T2 signal consistent with myocarditis. The patient was treated with non-steroidal anti-inflammatory drugs (NSAIDs) and azithromycin and had complete resolution in symptoms. He was exercise-restricted for six months.

Myocarditis is a potentially fatal pathology, representing a significant cause of sudden death in young adults. Myocarditis can present with a broad spectrum of signs and symptoms as well as variable clinical severity. Bacterial causes of myocarditis are uncommon, with *Campylobacter* among the least common. *Campylobacter *gastroenteritis, however, is quite common worldwide. Extra-intestinal and cardiac manifestations are rare; thus, it is important to maintain a high index of suspicion. Due in part to its rarity, treatment for *Campylobacter-*associated myocarditis is not well established. Treatment for myocarditis, regardless of etiology, is largely supportive in nature. *Campylobacter*-directed antibiotics, such as azithromycin, have been used successfully in adolescents with *Campylobacter*-associated myocarditis. Non-steroidal anti-inflammatory drugs (NSAIDs) are frequently used for symptom control, though their use remains controversial. Activity restriction is recommended for six months to reduce the risk of sudden cardiac death.

Myocarditis is an important cause of sudden death in young adults and is a rare extra-intestinal manifestation of *Campylobacter* bacterial gastroenteritis. Pediatric and adult providers should be aware of this presentation and its pathophysiology. They should also utilize a multi-modal workup, aggressive supportive care, appropriate subspecialty consultation, and appropriate antibiotics for patients with diarrheal illness and a high clinical suspicion for extra-intestinal involvement, such as myocarditis.

## Introduction

Myocarditis represents a significant cause of sudden death in young adults and thus is important for physicians to recognize and understand. Chest pain is commonly noncardiac in adolescent patients, yet cardiac etiologies of chest pain, such as myocarditis, often represent crucial “can’t miss” diagnoses. Myocarditis can present with a broad spectrum of signs and symptoms as well as variable clinical severity. Bacterial causes of myocarditis are rare, with *Campylobacter* among the least common. *Campylobacter* gastroenteritis is quite common worldwide, though extra-intestinal manifestations such as myocarditis are rare. Because intestinal *Campylobacter* infections are common, it is important to maintain a high index of suspicion for the rare complications of the disease, given that these manifestations can be severe. We present a case of a teenage patient presenting with chest discomfort, found to have an extra-intestinal manifestation of *Campylobacter* gastroenteritis in the form of myocarditis. We additionally briefly review common management and treatment strategies.

## Case presentation

A 17-year-old male with a history of supraventricular tachycardia (status-post radiofrequency ablation eight years prior) presented in June to a local emergency department with chest discomfort and chest pain on inspiration. He first noticed symptoms two days prior, after football practice, with subsequent development of headache, myalgia, vomiting, and diarrhea. He had no recent upper respiratory symptoms, including cough, rhinorrhea, or congestion, and denied recent immunizations. He had been previously diagnosed with COVID-19 twice, most recently six months prior to presentation. The patient lived in a mobile home community in rural Ohio, had recently been swimming in a freshwater lake and had eaten home-prepared deer meat. His father, who had also eaten the deer meat, had diarrhea as well.

Vital signs on presentation included a blood pressure of 115/77, pulse of 90, respiratory rate of 19, temperature of 98.7F (later Tmax of 100.7F), and oxygen saturation of 99% on room air. On exam, the patient was obese with a BMI of 48.5, had a regular pulse, brisk capillary refill, no peripheral edema, no jugular venous distension, no chest wall tenderness, and normal heart sounds without murmurs or rubs. His lungs were clear, and his breathing was unlabored. There was no evidence of hepatosplenomegaly, and his abdomen was soft and nondistended. 

Laboratory workup (Table 1) revealed elevated high-sensitivity troponin at 15,857 ng/L (ref range <20), >22,000 ng/L three hours later, and peak troponin of 37.7 ng/mL (ref range < 0.029) on arrival to our facility (different scale/assay than referring hospital) which downtrended thereafter. Additional labs showed leukocytosis of 16.1 x 10^9^/L, elevated ESR of 103 mm/h (0-15), CRP of 14.1 mg/dL (0-1.0), and CK of 1451 u/L (21-232). Basic metabolic panel (BMP)/chemistry and brain natriuretic peptide (BNP) were within normal limits. A gastrointestinal polymerase chain reaction (PCR) panel detected *Campylobacter *spp., and stool culture yielded *C. jejuni*. Nasopharyngeal respiratory viral PCR testing did not detect any targets. Blood culture yielded no bacterial growth and microbial cell-free DNA (Karius®), which evaluates for over 1000 bacteria, viruses, and fungi, did not detect any organisms in the blood.

**Table 1:** Laboratory values. PCR: polymerase chain reaction.

Laboratory study	Patient’s laboratory value	Reference range
Troponin (high sensitivity)	15,857 ng/L (presentation), >22,000 ng/L (three-hour interval)	<20 ng/L
Troponin (conventional)	37.7 ng/mL (peak)	<0.029 ng/mL
White blood cell count (WBC)	16.1 K/mcL	4.5-13.0 K/mcL
Erythrocyte sedimentation rate (ESR)	103 mm/h	0-15 mm/h
C-reactive protein (CRP)	14.1 mg/dL	0-1.0 mg/dL
Creatine kinase (CK)	1451 u/L	21-232 u/L
Gastrointestinal PCR panel	*Campylobacter *spp.	Negative
Stool culture	Campylobacter jejuni	Negative
Nasopharyngeal respiratory viral PCR	Negative	Negative
Blood culture	No growth	No growth
Microbial cell-free DNA (Karius®)	Negative	Negative

**Figure 1:**
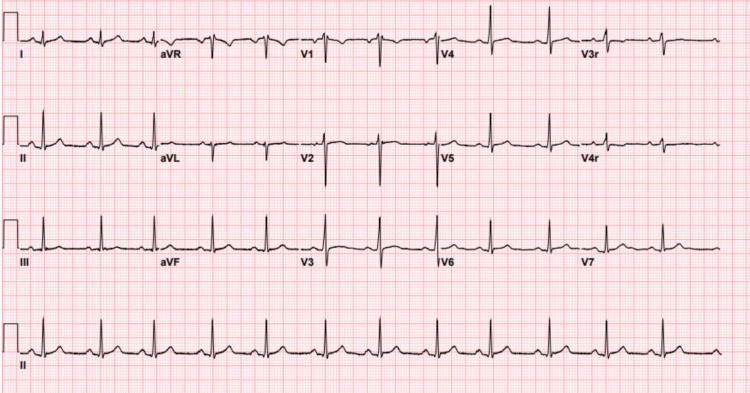
Patient's ECG on presentation demonstrating a normal ST pattern with an age-appropriate T wave pattern.

**Figure 2:**
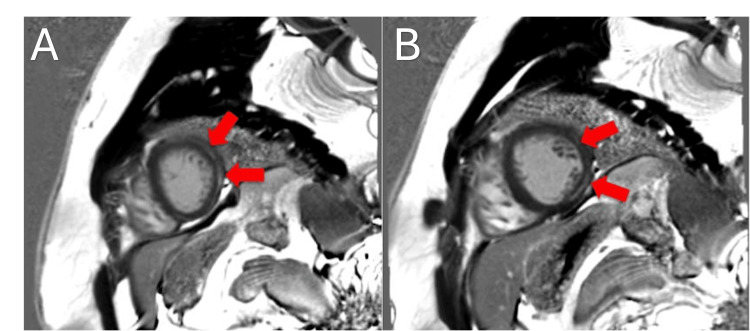
Cardiac MRI (CMRI) obtained during hospitalization. Panels (A) and (B) represent short-axis CMRI slices at different levels. Red arrows indicate increased T2 signal in the mid-anterolateral and inferolateral walls corresponding with areas of subepicardial delayed enhancement.

Outside of the hospital, chest X-rays and CT angiography were negative for acute cardiopulmonary processes and pulmonary embolism, respectively. The ECG presented to our institution showed a normal sinus rhythm with normal ST segments and T wave pattern, as shown in Figure 1. Bedside point-of-care transthoracic echo (performed by the cardiology fellow on-call) demonstrated normal biventricular size and function. Cardiac MRI (CMRI) revealed an increased T2 signal in the mid-anterolateral and inferolateral walls corresponding with areas of subepicardial delayed enhancement, as seen in Figure 2. CMRI also demonstrated mild biventricular dilation but normal biventricular systolic function.

## Discussion

Diagnosis

Given the presentation of pleuritic chest pain, headache, myalgia, diarrhea, and vomiting with elevated troponin and inflammatory markers, the initial differential diagnosis included bacterial/viral gastroenteritis, viral syndrome, pericarditis, myocarditis and less likely acute coronary syndrome or pulmonary embolism with heart strain. Ultimately, the clinical presentation, elevated troponin without ECG evidence of ischemia, *C. jejuni* positive stool, and CMRI evidence of myocardial inflammation suggested a diagnosis of clinically suspected myocarditis, most likely due to *Campylobacter jejuni *infection.

Myocarditis diagnosis and etiologies

Myocarditis is broadly defined as inflammation of the heart muscle and is a significant cause of sudden death in older children and young adults [1,2]. The presentation can be fulminant but often involves sub-clinical or mild prodromal symptoms [2,3]. Definitive diagnosis requires endomyocardial biopsy, though this is rarely performed, and "clinically suspected myocarditis" is more often diagnosed by clinical presentation, ECG, and imaging findings [4]. The European Society of Cardiology (ESC) outlines criteria for clinically suspected myocarditis, including at least 1 specified "clinical presentation" and 1 other “diagnostic criteria”. Clinical presentations outlined by the ESC criteria include acute chest pain, new-onset or worsening dyspnea/fatigue at rest or with exercise, palpitations and/or unexplained arrhythmia symptoms and/or syncope, aborted sudden cardiac death, or unexplained cardiogenic shock. ESC diagnostic criteria include ECG features, troponin (or another myocardiocytolysis marker) elevation, functional or structural abnormalities on cardiac imaging, or features of myocarditis on CMRI [4]. In asymptomatic patients, two diagnostic criteria are required [4]. It is noteworthy that our patient’s ECG was normal despite later demonstrating myocardial inflammation on CMRI, illustrating that ECG is not sufficient for excluding myocarditis.

Myocarditis can be infectious, toxin-related, hypersensitivity-induced, or related to systemic disease [3]. Infectious causes in North America and Europe are predominantly viral, with adenovirus and enterovirus being the most often identified pathogens [3]. Bacteria, fungi, helminths, protozoa, rickettsia, and spirochetes, though less common, can cause myocarditis as well [3,5]. Bacterial myocarditis is generally uncommon, with Campylobacter-associated myocarditis being additionally uncommon within bacterial causes; however, cases have been reported [6,7]. Conversely, Campylobacter is a common cause of bacterial gastroenteritis worldwide, illustrating the importance of understanding less common but potentially serious extra-intestinal manifestations [8]. The pathogenesis of *Campylobacter*-associated myocarditis remains uncertain, but potential mechanisms include direct bacterial invasion of cardiac tissue, release of bacterial toxins, and circulating immune complexes [5,9].

Treatment and management

Campylobacter gastroenteritis is generally self-limited, and antibiotics are not routinely recommended. A 2006 meta-analysis of 11 randomized controlled trials indicated that intestinal symptoms subsided 1.32 days earlier with antibiotics than with placebo [10]. The benefits of modestly shortened illness are likely outweighed by the risks of antimicrobial resistance in low-risk populations [10]. For higher-risk populations, such as immunocompromised patients or those with extra-intestinal manifestations (such as myocarditis), antibiotic therapy is reasonable [10]. Treatment with azithromycin (10 mg/kg/day, for three days) or erythromycin (40 mg/kg/day, in four divided doses, for five days) usually eradicates the organism from stool within two to three days. The recommended treatment duration for gastroenteritis is three to five days [11]. In a group of eight adolescent *Campylobacter*-associated myocarditis cases, six were treated with antibiotics and experienced symptom resolution, one was treated without antibiotics and recovered, and one individual treated without antibiotics died [7,12]. Though anecdotal, the only reviewed case of an adolescent death from *Campylobacter*-associated myocarditis did not receive antibiotic therapy [12].

When myocarditis is suspected, treatment is initially supportive regardless of the suspected etiology. Patients should be monitored for atrial and ventricular arrhythmias, hemodynamic status should be assessed, and inotropic support initiated promptly if indicated. If severe disease or cardiogenic shock is present, providers should consider the potential need for mechanical circulatory support such as extracorporeal membrane oxygenation (ECMO) or ventricular assist device and transfer to a center capable of such interventions early in the disease course. Antiviral and immunotherapy, including intravenous immunoglobulin (IVIG) and corticosteroids, may have a role in myocarditis treatment, but the approach is nuanced based on etiology, and pediatric data is lacking, making broad-based recommendations challenging [13]. When applicable, early involvement of infectious disease and rheumatology specialists is reasonable to aid in the use of these agents.

NSAID (including aspirin) use in myocarditis is controversial, with some animal data suggesting harm, while small studies in humans fail to demonstrate the same harm [14,15]. Although NSAIDs are indicated and frequently used in isolated pericarditis and other inflammatory diseases, animal models of myocarditis demonstrate increased inflammation, myocardial necrosis, and overall mortality when compared to placebo, particularly if utilized in the acute phase of illness [14]. Proposed mechanisms for harm associated with NSAID in myocarditis include decreased viral clearance, exaggerated cytotoxic response, and coronary artery spasm [14]. NSAID use may also counteract the beneficial mechanisms of angiotensin-converting enzyme inhibitors (ACEi)/angiotensin II receptor blocker (ARB) therapy seen in chronic heart failure management, further supporting careful and judicious NSAID use in myocarditis, particularly in the absence of pericardial inflammation and effusion [13]. Interestingly, a recent small retrospective case-control study in humans suggested no clear deleterious effects of NSAID use on mortality, in-hospital complications, or left ventricular systolic function [14,15]. Though not reaching statistical significance, two of the 114 patients in the study died and both received NSAIDs for myopericarditis [15].

Another pillar of myocarditis management is exercise restriction throughout the acute phase of illness and for at least six months from initial symptom onset. The rationale for restriction is to reduce the risk of sudden cardiac death [16]. The ESC recommends ECG, echocardiography, and exercise testing in addition to history and physical exam at follow-up within six months from presentation. When six months have passed from symptom onset, symptoms have completely resolved, and testing demonstrates normal left ventricle (LV) function with no arrhythmias or inducible symptoms with exercise, the patient may return to competitive sports or the previous level of exercise [16]. 

Patient course

Our patient did not develop impaired cardiac function or arrhythmias while admitted and his chest pain improved with initiation of NSAID therapy (naproxen 500 mg BID). He was prescribed azithromycin 500 mg for three days and was discharged in stable condition, with improvement in his symptoms. Troponin downtrended from a peak of 37.7 to 7.1 ng/mL. At the cardiology follow-up four days after discharge, he had a resolution of his chest pain and gastrointestinal symptoms. He had completed his azithromycin course, his previously scheduled NSAID was stopped, and he was exercise-restricted for six months. Troponin was rechecked at a three-month follow-up and normalized to undetectable levels.

## Conclusions

Myocarditis is a rare but potentially life-threatening sequela of *Campylobacter* gastroenteritis. *Campylobacter* gastroenteritis is common worldwide, even though resultant myocarditis is rare. Clinicians must maintain a high index of suspicion for extra-intestinal manifestations such as myocarditis in patients with diarrheal illness and cardiac or hemodynamic concerns. Antibiotic treatment is reasonable for patients with myocarditis secondary to bacterial gastroenteritis. NSAIDs should be used thoughtfully in myocarditis, particularly in cases of severe disease. Consultation with subspecialty colleagues is appropriate and can aid in managing patients with complex or atypical myocarditis. 
